# Supplementary material for: Dynamic transcriptomic profiles of zebrafish gills in response to zinc supplementation
Source: BMC Genomics. 2010 Oct 11;11:553. doi: 10.1186/1471-2164-11-553 (PMC3091702; doi:10.1186/1471-2164-11-553)
Supplement: Additional file 2 — Interactive Direct Interaction Network representing the molecular interactions between zinc, copper, iron, calcium and proteins encoded by transcripts changed by zinc supplementation. Mini web-site containing index.html and hyperlinked pages in subdirectory describing a Direct Interaction Network automatically generated based on curated interactions contained within the proprietary PathwayArchitect database. Ovals represent proteins and the circles symbolize metal ions. Objects are coloured by their abundance in zebrafish at the time-point they were significantly different from the control is a scale from -4 fold (dark green) to +4 fold (dark red). Where significant differences were found at more than one time-point, the colour overlay shows expression at the first instance. Dark blue squares denote 'binding', and light blue squares 'expression'; green squares stand for 'regulation', green diamonds for 'metabolism', and green circles for 'promoter binding'. Arrow heads indicate directionality of the interaction where annotated. All nodes and edges can be further interrogated by selecting the relative area of the image. [file 1471-2164-11-553-S2.zip › PathwayArchitect Zn xs DIN/2094786.html]

# PROMOTER BINDING:

|  |  |
| --- | --- |
| Type | PROMOTER BINDING |
| Effect | None |


---

|  |  |
| --- | --- |
| Score | 0 |


---

|  |  |
| --- | --- |
| Reference Count | 1 |


---

|  |  |
| --- | --- |
| Mechanism | Unknown |


---

|  |  |
| --- | --- |
| Reference:0 || PMID | 15546613 |
| Year | 2004 |
| SourceID | 301561 |
| Species | Human |
| Experimental Condition | in-vivo |
| Journal | Mol Cell |
| Description | An interaction between c-Jun and NCOA4 promoter was demonstrated by chromatin immunoprecipitation and promoter array hybridization. BT474 human breast cancer cells treated with cisplatin were fixed with formaldehyde. Protein-crosslinked DNA was enriched by immunoprecipitation with anti-phospho-c-Jun (Ser63) antibodies. Crosslinking was reversed, and the enriched DNA and the control genomic DNA from whole cell extract were differentially labelled with Cy3 or Cy5 by ligation-mediated PCR. Test and control DNA were hybridized to a promoter array containing the promoters (1000 bp upstream and 500 bp downstream from transcription initiation site) of 3,083 human genes. The ratio of test to control DNA bound to each spot was determined from 3 replicate experiments and a p-value was calculated from the replications. Results were considered positive if the p-value was less than 0.05 and a fold change of greater than 1.5. Supplementary Table S3. |
| Detection Method | cross-linking studies |
| Source | BIND |
  |


---

|  |  |
| --- | --- |
